# Reply to Comment on “In vivo flow cytometry reveals a circadian rhythm of circulating tumor cells”

**DOI:** 10.1038/s41377-021-00625-3

**Published:** 2021-09-17

**Authors:** Xi Zhu, Yuanzhen Suo, Yuting Fu, Fuli Zhang, Nan Ding, Kai Pang, Chengying Xie, Xiaofu Weng, Meilu Tian, Hao He, Xunbin Wei

**Affiliations:** 1 State Key Laboratory of Oncogenes and Related Genes, Shanghai Cancer Institute, Med-X Research Institute and School of Biomedical Engineering, Shanghai Jiao Tong University, Shanghai, 200030 China; 2 Biomedical Pioneering Innovation Center, Peking University, Beijing, 100871 China; 3 School of Life Sciences, Peking University, Beijing, 100871 China; 4 School of Instrument Science and Optoelectronics Engineering, Beijing Information Science & Technology University, Beijing, 100192 China; 5 Biomedical Engineering Department, Peking University, Beijing, 100081 China; 6 Key Laboratory of Carcinogenesis and Translational Research (Ministry of Education/Beijing), Peking University Cancer Hospital & Institute, Beijing, 100142 China

**Keywords:** Biophotonics, Imaging and sensing

Dear Editor,

We thank Niedre et al. for their correspondence regarding our recent paper^1^. They proposed a point that the temporal distribution of circulating tumor cells (CTCs) monitored by diffuse in vivo flow cytometry in a multiple myeloma mouse model in their previous work^2^ might be different from our results. Niedre et al. claimed that CTC detection statistics deviated from Poisson but did not found circadian variations in CTC numbers in a multiple myeloma mouse model. They also cite another literature by Juratli. et al.^3^, in which the authors reported that CTC numbers did not always correlate with tumor size during cancer progression. However, by establishing an orthotopic mouse model of prostate cancer and utilizing the technology in vivo flow cytometry (IVFC), we found CTCs exhibited bursting activity and daily oscillation in an orthotopic model of prostate cancer^1^.

We and Williams et al.^2^ both used IVFC to analyze fluctuations of CTC counts that were genetically fluorescently labeled and detected. Williams et al.^2^ found short-term dynamics of CTCs on 31 days after inoculation. The variability in CTC detection rates was higher than predicted by Poisson statistics. In our work, CTCs at different stages of cancer were analyzed and found with different distributions at early and advanced stages. At the early stage, CTCs were quite rare. The CTC occurrence deviated significantly from a Poisson process. CTCs exhibited stochastic bursting activity at early stages, which could be the reason for the non-Poisson distribution. However, at the advanced stage of prostate cancer, the bursting activity of CTCs decreased and the occurrence of CTCs obeys Poisson statistics. This difference between our work and ref. ^2^ may be induced by different CTC levels in different tumor models. More importantly, our major finding is the circadian variation of CTC counts.

The circadian variation of CTC counts revealed in our work is based on the orthotopic mouse model of prostate cancer, a solid tumor model. The multiple myeloma model used by Williams et al.^2^ was a hematologic malignancy model. The microenvironments of solid tumors and hematologic cancers (e.g., extracellular matrix and immune cells) are different^4,5^. It is well known that the trafficking of immune cells and cytokines in the bone marrow exhibits dramatic circadian rhythm^6–8^. However, the immune system is severely damaged in SCID mice in the work of Williams et al.^2^. Considering the dissemination of malignant plasma cells is regulated by immune cells and cytokines in the bone marrow^6,9–11^, we speculate the circadian release of CTCs in the bone marrow of SCID mice might be lost. It is worth noting that Paiva et al. reported circadian distribution of CTCs in patients with multiple myeloma^12^. This result suggests that the circadian rhythm of CTC counts varied among different species and cancers. Further studies using immune-competent mice to study the daily fluctuation of CTCs in hematological malignancies may help to elaborate on this issue.

The paper cited by Williams et al. in the Commentary reported that CTC counts did not always correlate with the primary tumor size^3^, which was different from most studies in mice and patients^13–15^. Juratli et al. observed the phenomenon about the short-term fluctuation of CTCs but did not find deeper insight into the statistical information^3^. Our study reveals the regulation of CTC release during different stages of cancer in detail.

For the record, we initially submitted our paper to *Light: Science & Applications* on 1st November 2020 before the publication of Prof. Niedre’s paper. Our primary findings on the non-uniform distribution and circadian variation of CTCs had already been presented in the 14th International Conference on Photonics and Imaging in Biology and Medicine (PIBM) in 2017 and SPIE BiOS in 2020, respectively. We are happy to cite Prof. Niedre’s papers in further studies.

In conclusion, according to all the analyses above, it should be noted that the tumor model types play an essential role in CTC release. In our orthotopic solid tumor model, the circadian rhythm of biological factors that can regulate tumor cell dissemination is involved to induce the daily fluctuation of CTCs. Although further studies are required to address whether the circadian rhythm of CTCs is common in all cancer types or just specific to several cancer types, evaluation of currently available data suggests that the dissemination of tumor cells in prostate cancer may be regulated by circadian rhythm.
